# The Effect of Spinal Muscle Fatigue and Psychosocial Factors on Pressure-Pain Threshold in Healthy Adults

**DOI:** 10.1155/2023/7336477

**Published:** 2023-01-25

**Authors:** Susan Mais, Jo Armour Smith

**Affiliations:** Department of Physical Therapy, Chapman University, 9401 Jeronimo Road, Irvine 92618, California, USA

## Abstract

**Objective:**

Pain sensitivity decreases following isometric exercise. It is not clear whether this exercise-induced hypoalgesia (EIH) occurs to the same extent in men and women. It is also unclear if the effect is systemic or local to the exercised musculature. The aim of our study was to investigate whether fatiguing isometric exercise of the spinal and hip extensors would result in increased pressure pain threshold (PPT) at sites local to and remote from the exercised muscles in healthy men and women and whether there is a relationship between central sensitization, psychosocial factors, and PPT.

**Subjects:**

35 healthy adults (age 27.1 ± 4.5 years, 22 women).

**Methods:**

This was a within-subjects cohort study. Participants completed questionnaires quantifying central sensitization, pain catastrophizing, sleepiness/insomnia, anxiety, and depression. PPT was assessed at the lumbar and thoracic paraspinals, hamstrings, gastrocnemius, wrist, and third digit before and immediately after participants performed the Biering–Sorensen test to failure.

**Results:**

PPT increased postexercise in the thoracic paraspinals, hamstrings, and gastrocnemius in men and women and in the lumbar paraspinals in men only but did not change at the wrist and digit sites. A lower average PPT at baseline was associated with a higher central sensitization scores. A greater increase in average PPT postfatigue was significantly associated with higher average PPT at baseline.

**Conclusions:**

Exercise-induced hypoalgesia occurs at sites overlying the muscles involved in fatiguing exercise, but not at remote sites, and is more evident in males than females. The magnitude of EIH depends upon baseline PPT. Even in healthy individuals, greater central sensitization is associated with lower baseline PPT.

## 1. Introduction

An individual's pain sensitivity is affected in the short term and long term by multiple factors. Pain sensitivity can be assessed using the measurement of pressure-pain threshold (PPT). This is a simple quantitative sensory test of pain that is often used in research and clinical practice [1–4]. The PPT is defined as the minimum pressure applied to anatomical site that results in an individual perceiving the mechanical stimulus as pain [5]. The measurement of PPT is accurate, valid, and reproducible [6, 7]. Women typically display a lower PPT than men [8]. Individuals with persistent musculoskeletal pain disorders such as recurrent or chronic low back pain also demonstrate reduced pain thresholds when measured using PPT [9–11]. This reduced PPT may occur at the location of the clinical symptoms, suggestive of peripheral sensitization mechanisms, and also at locations that are not anatomically or neurophysiologically related to the symptoms, suggestive of supraspinal mechanisms [2, 10–12]. To understand the contribution of short- and long-term factors to localize and widespread pain sensitivity in individuals with persistent pain disorders, it is critical to first examine potential contributing factors in asymptomatic men and women.

Pain sensitivity is modulated in response to exercise [13]. In the short term, isometric muscle exercise appears to decrease pain sensitivity [3, 9, 14, 15]. This is called exercise-induced hypoalgesia (EIH). The greatest change in pain threshold occurs in response to long-duration, low-intensity isometric contraction that is maintained until failure [16]. However, it is unclear if the reduction in pain sensitivity following muscle fatigue is local to the affected muscle or if it is a generalized response that also occurs at sites remote to the fatiguing exercise [3]. The Biering–Sorensen test is a long-duration, low-intensity isometric task that is often used to quantify the endurance of the spinal and hip extensor musculature in individuals with and without low back pain [17, 18]. Previous preliminary work investigating the effect of the Sorensen test on PPT in the spinal extensors and remote sites suggested that the extent of EIH at remote locations is greater in women [3]. However, in this previous study, the Sorensen test was held for a standardized duration, and so the muscles may not have been fatigued to failure. Sex differences in the local and systemic hypoalgesic response to fatiguing exercise may depend on testing location as conflicting results across studies suggest that these differences are muscle specific [3, 19].

In the short and long terms, pain sensitivity can increase because of the heightened responsiveness of nociceptive neurons to normal or subthreshold afferent input. This phenomenon is known as central sensitization [20]. While central sensitization is a neurophysiological mechanism that cannot be directly measured in vivo, signs and symptoms such as widespread increased pain sensitivity suggest its presence [21]. Central sensitization is known to contribute to many persistent pain disorders [22]. Recently, it has been recognized that central sensitization may also be elevated in individuals who do not have persistent pain. Elevated central sensitization may in fact be a precursor to the development of clinical pain [22, 23]. It is not known if central sensitization influences pain sensitivity in nonclinical populations or if it alters the extent of hypoalgesia following isometric exercise. Clinically, central sensitization can be assessed using a combination of quantitative sensory testing and self-report measures. One such self-report measure is the Central Sensitization Inventory (CSI) [24], which quantifies the presence of biological and psychological symptoms and characteristics that are associated with central sensitization syndromes [24]. It is not clear, however, how quantitative measures such as PPT and self-report measures such as the CSI are related [25, 26].

Multiple psychosocial factors are associated with pain sensitivity. These include depression, anxiety, pain catastrophizing, and sleep quality. Depression and increased pain sensitivity frequently occur together [27], but the mechanism underlying this relationship is unclear [28]. Evidence also suggests that in individuals with and without persistent pain, elevated anxiety is associated with decreased PPT [1, 12]. Pain catastrophizing has been associated with a number of indicators of pain sensitivity in the context of experimental pain testing paradigms, both among healthy, pain-free participants and individuals with persistent pain disorders [29–32]. There is also increasing evidence of a relationship between sleep quality and an individual's pain experience [33]. In the short term, healthy individuals have increased pain sensitivity following experimentally-induced sleep deprivation [34, 35]. Long-term sleep disruption is also associated with increased pain sensitivity in individuals with and without persistent pain disorders [36]. However, it is unclear if any of these psychosocial factors influence EIH.

The aim of our study was to investigate whether performing a long-duration, low-intensity fatiguing exercise to failure would result in increased PPT at local and remote sites in healthy men and women and whether there is a relationship between central sensitization, psychosocial factors, and PPT in healthy individuals. It was hypothesized that (1) the fatiguing isometric exercise would produce an increase in PPT at both local and remote sites; (2) the increase in PPT in response to fatiguing exercise would be more pronounced in women than in men; and (3) impaired sleep quality, and increased anxiety, pain catastrophizing, and central sensitization would be associated with lower PPT in healthy adults at baseline and would be associated with a smaller extent of change in PPT in response to fatiguing exercise.

## 2. Materials and Methods

### 2.1. Participants

Thirty-five healthy adults (mean age of 27.1 ± 4.5 years, 22 women and 13 men) participated in the study. Sample size exceeded the minimum necessary to detect a main effect of fatigue and the interaction effect of fatigue and sex with a power of 0.90 and an alpha of 0.05 (*n* = 22, *G*^*∗*^Power version 3.1.9.7 [3, 37]). Participants were recruited via word of mouth and study flyers at an academic institution and included faculty members, staff, and students. Participants were eligible for inclusion if they were aged between 18 and 60 years. Participants were excluded if they were currently experiencing any back or leg pain, had any history of back pain requiring treatment, or a change in activity for more than one week, if they were currently using any form of analgesic medication and if they had any history of neurological or cardiovascular diseases. Participants gave written consent prior to participating. Chapman University's Institutional Review Board approved this study prior to its commencement.

### 2.2. Psychosocial Factors

All testing was conducted in the same teaching laboratory space on a consistent day of the week/time of day. Participants completed the Central Sensitization Inventory (CSI), the Pain Catastrophizing Scale (PCS), the Karolinska Sleepiness Scale (KSS), the Insomnia Severity Index (ISI), and the Hospital Anxiety and Depression Scale (HADS) prior to PPT assessment. The CSI is a widely used questionnaire that is valid and reliable for assessing central sensitization [24, 38]. Part A of the CSI contains 25 questions identifying symptoms associated with central sensitization and has a maximum score of 100. Part B assesses if the individual experiences any disorders associated with central sensitivity, such as fibromyalgia and temporomandibular disorder. The PCS is a reliable and valid measure of pain catastrophizing and includes the domains of pain magnification, rumination, and helplessness, with a maximum score of 52 [39]. The KSS is used to estimate the state-sleepiness of participants, with a score range from 1 to 9 [40]. It has high validity and correlates with EEG and behavioral indicators of sleepiness [41]. The ISI is a valid and reliable tool to assess perceived insomnia, with scores ranging from 0 to 28 [42]. The HADS is a validated self-report measure of psychological status, encompassing depression and anxiety subscales (HADS-D and HADS-A, maximum scores of 21 for each subscale [43]).

### 2.3. Assessment of Pressure-Pain Thresholds

Prior to the PPT assessment, participants warmed up by walking on a treadmill at a speed of 2.8 mph for 5 minutes. Upon completion of the warm-up, participants quantified their perceived exertion during the walk on the 0–10 Rating of Perceived Exertion (RPE) scale [44, 45]. Participants also rated any pain/discomfort from 0 to 10 after the warm-up on a numeric pain rating scale.

**Figure 1:**
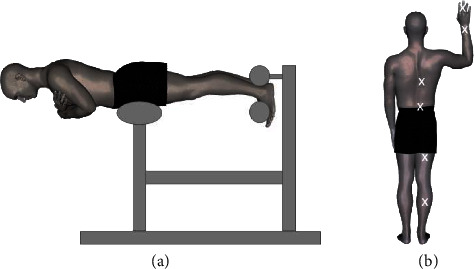
(a) Performance of the isometric fatiguing exercise (Sorensen test) on a roman chair. (b) Location of pressure-pain threshold assessment sites (note that the digit site was on the palmar surface of the finger).

The sites for PPT assessment were palpated and marked with an indelible pen so that the location of each site was identical prefatigue and postfatigue. Pressure-pain threshold was assessed at the following sites on the participants' dominant sides (Figure 1): the erector spinae at the level of T9, 3 cm lateral to the spinous process (thoracic site); erector spinae at the level of L4, 2 cm lateral to the spinous process (lumbar site); the hamstrings halfway between the ischial tuberosity and the popliteal crease (hamstring site); the gastrocnemius one third of the distance between the calcaneus and the popliteal crease (calf site); the palmar aspect of the middle phalanx of the third digit (digit site); and the dorsal wrist at the midpoint between the ulnar and radial styloid processes (wrist site).

Pressure-pain threshold was measured utilizing a handheld pressure algometer with a 1 cm diameter circular application area (FDX Digital Force Gauge, Wagner Instruments, and CT). Increasing pressure was applied at a rate of approximately 0.5 kg/s. The same male experimenter performed all PPT assessments for the study and was blinded to the results. Participants were instructed on the testing procedure and were told to verbally indicate immediately when the sensation of pressure changed to pain. A practice trial at the dorsal wrist was administered to the participant while seated. Following this, PPT was assessed while the participant was prone on a plinth. Two PPT measurements were taken at each site, with 20 seconds between each measurement [3]. The order in which the sites were assessed was randomized across participants, but for each participant the order was the same before and after exercise.

### 2.4. Isometric Fatiguing Exercise

Following the baseline PPT measurements, participants completed the Biering–Sorensen test (Figure 1(a)). The Sorensen test fatigues the spinal extensor, latissimus dorsi, gluteus maximus, and hamstring muscles [46, 47]. Participants were positioned on a Roman chair, with the anterior superior iliac spines aligned with the edge of a pelvic pad. Participants were instructed to hold their body parallel to the ground with the arms crossed across their chest for as long as possible. To assist in monitoring the performance of the test, a plumb bob at the end of a lanyard was placed around the neck of each subject during the test. The researchers adjusted the lanyard so that the plumb bob was one inch above the seat of a standard-height chair placed in front of the participant. The test was concluded when the participant placed their hands back down onto the chair or if the plumb bob dropped to the chair, indicating failure to maintain the test position [48], and the duration of the test was noted. Immediately following completion of the Sorensen test, participants rated the intensity of exertion during the test using the same RPE scale and rated the intensity of any pain/discomfort during the test on the numeric pain rating scale. The PPT measurement was then repeated using identical methods to the prefatigue testing. Postfatigue PPT assessment for all sites was completed within 5 minutes of the end of the fatiguing exercise. This ensured that the postfatigue testing was applied during the window of time when the muscles were still measurably fatigued [49].

### 2.5. Statistics

Variables were checked for assumptions of normality and homoscedasticity. Variables that did not meet the assumptions of normality were log-transformed. Psychosocial characteristics were compared between males and females using independent *t*-tests and the chi-square test of independence. The effect of fatigue at each assessment site, and averaged across all assessment sites, was tested using a mixed model ANOVA with the main effect of fatigue (within subject effect), main effect of sex (between subject effect), and interaction effect of fatigue^*∗*^sex. In the case of a significant interaction, pairwise comparisons were made using paired or two sample *t*-tests with Bonferroni correction for multiple comparisons.

Linear relationships between psychological variables and (a) the average PPT at baseline and (b) average change in PPT were examined using Pearson correlation coefficients. Statistical analyses were conducted in IBM® SPSS® statistical software (Version 26, IBM, Armonk, NY, USA). Level of significance was set at 0.05 for all tests.

## 3. Results

Participant demographic characteristics are shown in Table 1. The duration of the Sorensen test did not differ between sexes (female duration 126.6 ± 52.8 s; male duration 126.3 ± 64.8 s). There was also no difference between sexes for the rating of perceived exertion at the end of the test or discomfort reported at the end of the test (see Table 1, *p* > 0.05 for all comparisons). Female participants scored higher on the HADS (anxiety subscale) and tended to score higher on the CSI than males (see Table 1, *p*=0.035 and 0.052, respectively). Eight individuals (22% of the cohort) reported at least one disorder in Part B of the CSI. Six individuals reported one disorder. These were most commonly headache (*n* = 2), neck injury (*n* = 2), and anxiety (*n* = 2). Two individuals reported two disorders (chronic fatigue/temporomandibular joint disorder and headache/irritable bowel syndrome, respectively).

### 3.1. Baseline PPT and Change in PPT in Response to Fatigue

**Figure 2:**
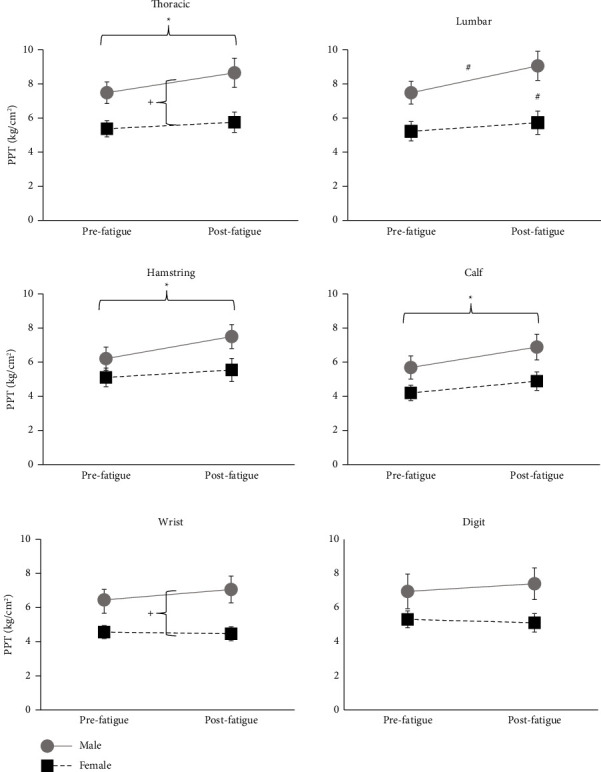
Pressure-pain threshold (PPT) prefatigue and postfatigue in male and female participants. ^*∗*^indicates significant main effect of fatigue. ^+^indicates significant main effect of group. ^#^indicates significant pairwise post hoc comparison for interaction between fatigue and group.

Data from one male participant were excluded due to his PPT exceeding the maximum possible pressure for the device at several sites. Group data from three sites were log-transformed to meet assumptions of normality (calf, wrist, and digit). Group data for all sites, prefatigue and postfatigue, are shown in Figure 2.

For pressure-pain threshold averaged across all sites, there was a significant main effect of fatigue (*F* = 22.276; *p*=0.001), as well as a main effect of sex (*F* = 6.002; *p*=0.020) and fatigue by sex interaction (*F* = 6.622; *p*=0.015). Bonferroni-corrected post hoc comparisons indicated that average PPT increased significantly postfatigue in males (*p*=0.012) but not in females. None of the other pairwise comparisons were significant.

Analysis of the individual PPT sites indicated that the effect of fatigue on PPT varied by sex and by testing site. For the thoracic site, PPT was significantly higher in both groups postfatigue (main effect of fatigue, *F* = 9.891; *p*=0.004). Thoracic PPT was also higher in males than in females prefatigue and postfatigue (main effect of sex, *F* = 7.709; *p*=0.009). At the lumbar site, there was a significant fatigue by sex interaction (*F* = 8.031; *p*=0.008). Bonferroni-corrected post hoc comparisons indicated that males had higher PPT than females postfatigue (*p*=0.024) and that there was a significant increase in PPT postfatigue in males (*p*=0.004) but not in females. At the hamstring site, PPT was higher postfatigue in both groups (main effect of fatigue *F* = 11.660; *p*=0.001). There was also a trend toward a significant fatigue by sex interaction (*F* = 4.111; *p*=0.051) but post hoc comparisons by sex were nonsignificant. At the calf site, PPT increased significantly postfatigue in both males and females (main effect of fatigue *F* = 20.866; *p*=0.001). There was a trend toward males having higher PPT at the calf (main effect of sex *F* = 3.964; *p*=0.055).

At the wrist, there was no effect of fatigue on PPT (main effect of fatigue *F* = 0.191; *p*=0.665). Males had a higher PPT at the wrist than females (main effect of sex *F* = 8.346; *p*=0.007). At the digit site, there was no main effect of fatigue (main effect of fatigue *F* = 0.359; *p*=0.554) or sex (*F* = 2.567; *p*=0.120). There was a significant fatigue by sex interaction (*F* = 4.581; *p*=0.041) but none of the post hoc comparisons were significant.

### 3.2. Relationships between Baseline PPT, Change in PPT, and Psychological Characteristics

Data from the PCS and the KSS were log-transformed to meet assumptions of normality. Relationships between baselines PPT, change in PPT, and psychological characteristics across the entire group and for each sex individually are shown in Table 2 and significant findings are reported below.

**Figure 3:**
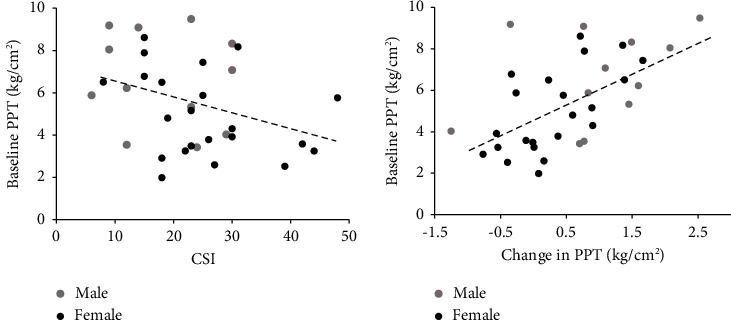
(a) Significant linear relationship between baseline pressure-pain threshold (averaged across sites, PPT) and score on the Central Sensitization Inventory (CSI); *r* = −0.352; *p*=0.041. (b) Significant linear relationship between baseline pressure-pain threshold (average across sites, PPT) and change in PPT postfatigue; *r* = 0.576; *p*=0.001.

For baseline PPT, greater central sensitization was significantly associated with lower average PPT at baseline (*r* = −0.352; *p*=0.041; Figure 3(a)). In males only, a greater history of insomnia was associated with lower PPT at baseline (*r* = −0.593; *p*=0.042).

For change in PPT following the Sorensen test, greater increase in average PPT postfatigue was significantly associated with higher average PPT at baseline (*r* = 0.576; *p*=0.001; Figure 3(b)). With the sexes considered separately, this relationship was only significant in females (*r* = 0.630; *p*=0.002). Greater pain catastrophizing was significantly associated with a smaller change in PPT in response to fatigue in females (*r* = −0.461; *p*=0.031).

## 4. Discussion

Our study demonstrates that isometric contractions held to failure result in an increase in pressure-pain threshold in healthy individuals at local muscle sites but not at sites that are remote to the fatiguing exercise. Contrary to our hypothesis, the increase in PPT in response to fatiguing exercise was more pronounced in men than in women. Importantly, individuals with higher PPT at baseline had the greatest increase in PPT in response to fatigue. Even in our healthy participants, elevated CSI scores were associated with lower baseline PPT. The influence of psychosocial factors on baseline PPT and fatigue-induced changes in PPT were sex-dependent.

Our findings confirm the hypoalgesic effect of isometric exercise. Earlier studies have indicated that reduced pain sensitivity in response to exercise is most pronounced following prolonged, low-intensity, isometric contractions. This suggests that recruitment of high-threshold motor units may be an important factor in the hypoalgesic response [14]. Mechanistic studies investigating the causes of EIH have predominantly focused on adaptations in response to chronic, whole-body aerobic exercise in animal models [13]. This work has highlighted the influence of endogenous opioid, serotonergic, and endocannabinoid systems. These mechanisms produce generalized pain inhibition. Other work probing localized and generalized responses to isometric exercise in humans has suggested that generalized EIH may occur as a result of increased blood pressure or altered attention, but some research study has also demonstrated that effects are largest in the contracting muscle, suggestive of a local or segmental effect [9]. In the present study, significant increases in PPT following the fatiguing exercise occurred at the sites overlying the spinal and hip extensor muscles. These muscles are known to fatigue during the Sorensen test [49, 50]. The local modulation of PPT in the fatigued muscles may be due to afferent inhibition in response to stimulation of sensory fibers during the fatiguing contraction [9]. In this study, the PPT at the calf site also increased following the Sorensen test. The contribution of the calf musculature to maintenance of the Sorensen test position has not previously been reported. However, it is likely that the gastrocnemius is active during the test to develop a knee flexor moment that keeps the heels stabilized against the test equipment. In our study, we did not observe generalized EIH, as there were no changes in PPT at the wrist and digit sites that were remote to the exercise. Previous work reported increased PPT at the thenar eminence following the Sorensen test [3]. We speculate that the amount of muscular tissue overlying remote sites may influence the extent of measured EIH due to differences in tissue thickness, tissue stiffness, and density of mechanoreceptors. Additionally, our findings are consistent with recent work suggesting that generalized EIH is less likely to occur following fatiguing isometric exercise of the back musculature in comparison with fatiguing exercise of the limb musculature [51]. The reasons for this are unclear but may be related to varying exercise intensity when exercising axial or limb muscles or differing muscle fiber types [51].

In contrast with earlier work using the Sorensen test paradigm [3], we found that the increase in PPT in response to fatiguing exercise occurred more consistently in male participants. Other studies using PPT have variously reported that EIH following isometric contraction is the same in men and women, that it is greater in women, or that it is greater in men [52–54]. The conflicting findings from these studies may in part be due to varying fatigue protocols [53]. In the present study, participants held the Sorensen position for as long as possible, until failure, rather than for a standardized length of time. This ensured that participants of all capabilities reached the limit of their muscle endurance. Although the duration of the hold time varied widely across individuals (20 seconds to 250 seconds), the average hold time (126 seconds) was greater than the standardized time used in the previous study (120 seconds) [3]. Our study supports earlier work suggesting that sex differences in EIH emerge primarily in response to more demanding exercise [53]. The smaller amount of EIH evident in female participants in our study may also have been due to the female group tending to have higher levels of anxiety and higher scores on the CSI than male participants. Given the heterogeneity in the evidence for the influence of sex on EIH and the potential interaction with psychological and physiological factors, future studies would benefit from large cohorts of male and female participants matched for psychosocial characteristics and should investigate EIH in response to a range of exercise intensities and durations.

Consistent with earlier work [55], baseline PPT was higher in males than females in this study. Factors that may account for the sex difference in pain sensitivity include the influence of sex hormones on nociception and differences in the function of the descending opioid system [55]. Sex differences in baseline PPT may also be due in part to differences in muscle size. Binderup et al. [56], proposed that as women have smaller muscle bulk on average, the size of the algometer probe head is relatively larger, leading to a lower PPT due to spatial summation. The results of our study lend support to this hypothesis, as we found PPT differences by sex at every site except for the 3rd digit, the only site where there is little muscle tissue.

Importantly, we found that the extent of EIH was linearly related to baseline PPT. Participants who demonstrated lower pain sensitivity at baseline had greater EIH. Vaegter et al. [57] have previously reported that increased pain sensitivity, and higher intensity of clinical pain at baseline, reduces exercise-induced hypoalgesia in individuals with back pain. Our findings demonstrate that the influence of baseline pain sensitivity on EIH extends to healthy individuals without pain. This indicates that a uniform hypoalgesic response to isometric exercise should not be assumed, even in healthy adults. We also demonstrate in this study that even in a population of healthy adults, higher scores on the Central Sensitization Index are associated with lower PPT at baseline. This is despite the fact that average scores on the CSI in our study were well below the threshold of 40 that has been suggested as the cutoff score for clinically relevant central sensitization in patient populations [58–60] and were also lower than scores previously reported in nonpatient cohorts [59]. The frequency of our participants reporting diagnoses associated with central sensitization (22%) was also far lower than that reported in patient populations [59]. Existing evidence for the relationship between scores on the CSI and quantitative measures of pain sensitivity in patient populations is mixed [26], but our study suggests an association between biopsychosocial characteristics identified by the CSI and pain sensitivity in healthy adults.

Other factors influencing baseline PPT or change in PPT postfatigue were sex-dependent. Females with higher pain catastrophizing scores demonstrated less hypoalgesia in response to the fatiguing exercise. Our findings build upon work by Naugle et al. [52], who reported that levels of pain catastrophizing were predictive of exercise-induced changes in the temporal summation of heat pain. The relationship between pain catastrophizing and reduced EIH may be a result of reduced descending opioid pain inhibition. It is not clear why the influence of pain catastrophizing on hypoalgesia was only evident in women, as, unlike in previous studies [52], there was no difference in pain catastrophizing scores between men and women in our cohort. Our study did not find significant relationships between sleep quality and baseline PPT or a change in PPT following fatiguing exercise in the combined group analysis. However, in males, we demonstrate that greater insomnia was associated with reduced PPT at baseline. The difference between our findings and previous work may be due to the fact that the KSS and ISI scales quantify state-sleepiness and the perceived level of current insomnia [41, 42], rather than long-term sleeping impairment. Additionally, levels of sleep disturbance reported in this study were below the threshold for clinically significant impairment [42] and were far lower than in studies involving experimental sleep deprivation.

There were some limitations to our study. The sample had an unequal distribution of male and female participants. Of note, changes in PPT in response to exercise were more pronounced in the smaller male cohort, indicating that our findings were not influenced by a lack of statistical power in the male group. Additionally, as in all studies investigating fatigue, it is difficult to prove objectively that muscle fatigue has occurred. We did not quantify the loss of force production following the Sorensen test, but loss of force production at the end of the test was indicated by the individuals' inability to continue maintaining the test position. In a previous study, using the same Sorensen paradigm in a cohort of young healthy adults, we confirmed with electromyography that fatigue occurs in the lumbar extensor muscles during the test [49]. We have also demonstrated that this fatigue persists for five to ten minutes after completion of the test, which was more than enough time to complete our PPT testing at all sites [49]. Finally, in this study, the mechanical stimuli were applied manually using a pressure dynamometer. It is possible therefore that the rate of pressure application varied slightly across testing repetitions. However, we minimized this potential confound by having the same trained tester apply the PPT stimuli to all participants.

### 4.1. Clinical Implications

The Biering–Sorensen test is a simple way to induce spinal and hip extensor fatigue and to assess EIH in healthy individuals and in clinical pain populations. Our findings of a significant relationship between PPT and scores on the CSI suggest that PPT testing may be a useful quantitative adjunct to self-report central sensitization questionnaires in the clinical setting. Isometric strengthening exercise is often recommended as an intervention for individuals with low back pain, and training that includes isometric extensor exercises such as “planks” or “bird dogs” is a common component of rehabilitation. However, since our study shows that the extent of EIH depends upon pain sensitivity at baseline, individuals with LBP with high baseline pain sensitivity may not respond in the same way to isometric exercise as back-healthy controls. Future studies should clarify if fatiguing isometric exercise reduces pain sensitivity in all patients with low back pain.

## 5. Conclusion

Isometric spinal and hip exercise held to failure produces a local hypoalgesic response. This occurs in both males and females, but to a greater extent in males. In healthy participants, there is greater EIH in those with lower pain sensitivity at baseline. In individuals without a clinical pain condition, a higher self-reported rating on the CSI is associated with a lower baseline PPT.

## Figures and Tables

**Table 1:** Participant demographic characteristics, psychosocial characteristics, and the Sorensen test outcomes.

	Females (*n* = 22)	Males (*n* = 13)	*p*
Age (years)	27.3 (5.2)	26.3 (3.2)	0.547
BMI (kg/m^2^)	23.2 (4.2)	25.3 (2.1)	0.096
Race, frequency			
American Indian/Alaska native	0	0	0.473
Asian	8	8	
Black/African America	0	0	
Native Hawaiian/other Pacific Islander	0	0	
White	10	4	
Mixed	3	0	
Unknown/not reported	1	1	
Anxiety, HADS-A	8.5 (2.5)	6.4 (3.3)	0.035^*∗*^
Depression, HADS-D	3.8 (2.5)	3.0 (1.4)	0.304
Central sensitization, CSI	25.5 (10.3)	18.6 (8.6)	0.052
Pain catastrophizing, PCS	9.1 (7.2)	8.5 (10.1)	0.851
Sleepiness, KSS	3.7 (1.7)	3.9 (1.3)	0.668
Insomnia, ISI	6.8 (4.6)	5.1 (3.7)	0.267
Sorensen duration (seconds)	126.6 (52.8)	126.3 (64.8)	0.989
Perceived exertion, 0–10	5.3 (1.7)	5.3 (1.5)	0.941
Pain/discomfort, 0–10	0.5 (1.3)	0.5 (1.2)	0.959

^*∗*^Statistically significant difference between males and females.

**Table 2:** Linear relationships between psychosocial factors, average baseline pressure pain threshold, and average change in pressure-pain threshold postfatigue.

		Average baseline	Average change
Anxiety, HADS-A	Entire sample	−0.201	−0.096
	Female	0.111	−0.042
	Male	−0.300	0.154

Depression, HADS-D	Entire sample	−0.175	−0.107
	Female	−0.069	−0.088
	Male	−0.277	0.062

Central sensitization, CSI	Entire sample	−0.352^*∗*^	−0.274
	Female	−0.329	−0.254
	Male	−0.136	−0.063

Pain catastrophizing, PCS	Entire sample	−0.321	−0.266
	Female	−0.285	−0.461^*∗*^
	Male	−0.391	−0.059

Sleepiness, KSS	Entire sample	−0.235	0.001
	Female	−0.350	−0.266
	Male	−0.236	0.379

Insomnia, ISI	Entire sample	−0.179	−0.228
	Female	0.101	−0.106
	Male	−0.593^*∗*^	−0.303

Average baseline	Entire sample	n/a	0.576^*∗*^
	Female	n/a	0.629^*∗*^
	Male	n/a	0.382

^*∗*^Statistically significant correlation.

## Data Availability

Data are available upon request.

## Supplementary Materials

Supplementary Materials Scatterplots are provided showing the relationship between pressure-pain threshold and the Hospital Anxiety and Depression Scale, Insomnia Severity Index, Pain Catastrophizing Scale, and Karolinska Sleepiness Scale. STROBE Statement-checklist of items that should be included in reports of observational studies.
