# Supplementary material for: The Effect of Spinal Muscle Fatigue and Psychosocial Factors on Pressure-Pain Threshold in Healthy Adults
Source: Pain Res Manag. 2023 Jan 25;2023:7336477. doi: 10.1155/2023/7336477 (PMC9891829; doi:10.1155/2023/7336477)
Supplement: Supplementary Materials — Scatterplots are provided showing the relationship between pressure-pain threshold and the Hospital Anxiety and Depression Scale, Insomnia Severity Index, Pain Catastrophizing Scale, and Karolinska Sleepiness Scale. STROBE Statement-checklist of items that should be included in reports of observational studies. [file 7336477.f1.zip › Supplementary Material.pdf]

SUPPLEMENTARY MATERIAL

Scatterplots showing relationship between pressure pain threshold (PPT) and psychosocial variables. Please see also Table 2 and Figure 3.

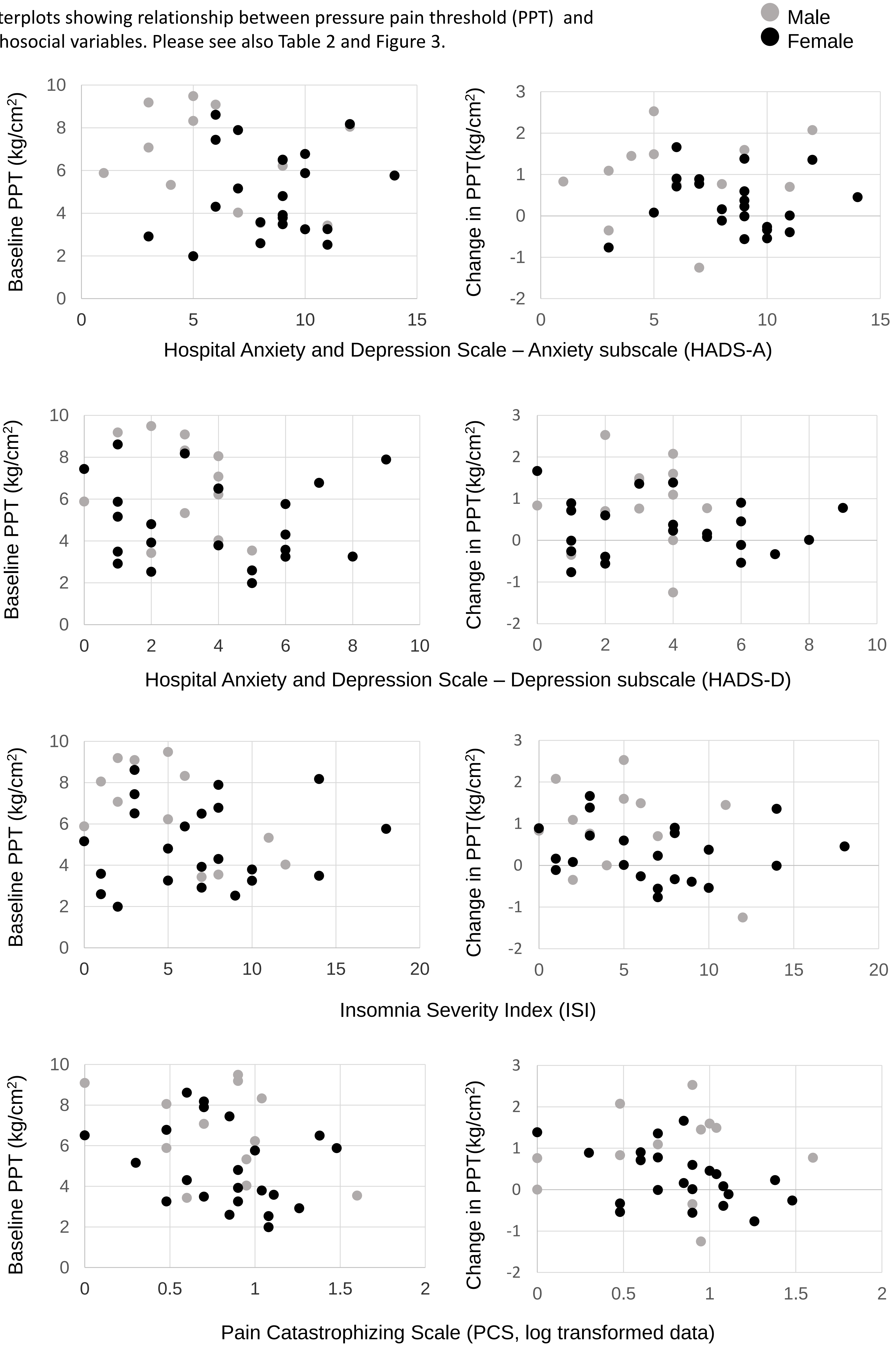

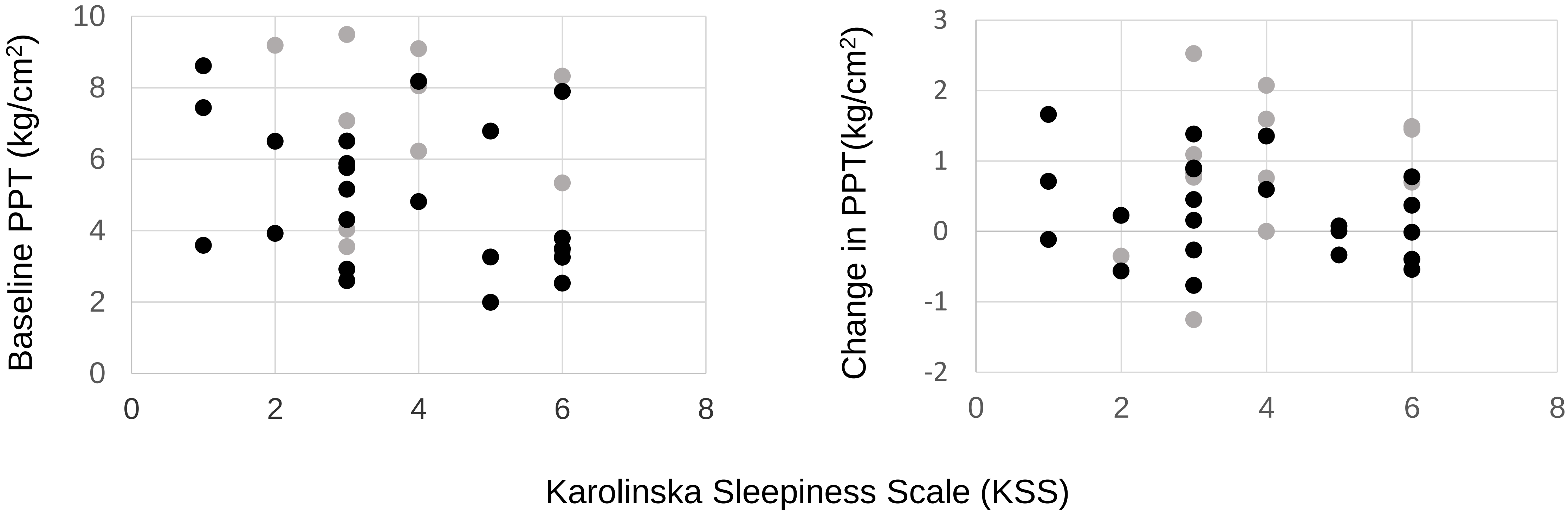
Baseline PPT (kg/cm²)
10
8
6
4
2
0
0
2
4
6
8
Change in PPT(kg/cm²)
3
2
1
0
-1
-2
0
2
4
6
8
Karolinska Sleepiness Scale (KSS)
